# Gd Metal–Organic Framework Thin Film for On-Chip Local Magnetic Refrigeration

**DOI:** 10.1021/acs.chemmater.4c00909

**Published:** 2024-08-20

**Authors:** Inés Tejedor, Dmitry E. Kravchenko, Jesús Gandara-Loe, Rob Ameloot, Ignacio Gascón, Olivier Roubeau

**Affiliations:** § Instituto de Nanociencia y Materiales de Aragón (INMA), CSIC and Universidad de Zaragoza, Zaragoza 50009, Spain; † Centre for Membrane Separation, Adsorption, Catalysis and Spectroscopy, KU Leuven, Celestijnenlaan 200F, Leuven 3001, Belgium

## Abstract

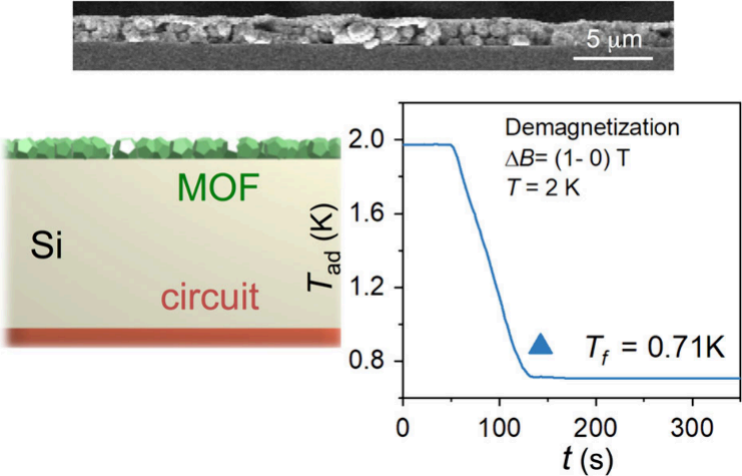

Dense metal–organic frameworks with high spin paramagnetic nodes are competitive materials for cryogenic magnetic refrigeration, particularly in applications for which local cooling is advantageous. We focus on obtaining thin films of gadolinium formate, which has a large volumetric magnetocaloric effect. Continuous and homogeneous deposits of gadolinium formate are successfully formed on silicon by means of aerosol jet printing, with control over the film thickness from 0.35 μm up to 2.5 μm. The excellent cooling power of the deposits is evidenced via direct measurements of the cooling of a 200 μm silicon wafer down to sub-K temperatures by a single demagnetization from 1 T and 2 K, thereby demonstrating the potential of this approach for on-chip local magnetic refrigeration.

## Introduction

Cryogenic magnetic cooling is a relatively mature technology^1^ for reaching sub-K temperatures, an alternative to the ^3^He–^4^He refrigeration process that remains under the threat of potential shortage.^2^ Cryogenic magnetic cooling relies on materials having isolated paramagnetic ions with high spin and low magnetic anisotropy, thereby possessing a high magnetocaloric effect (MCE).^3^ MCE is defined as the changes in adiabatic temperature (Δ*T*_ad_) or in magnetic entropy (Δ*S*_m_) of a material resulting from a change in magnetic field. Molecular-based materials have been proposed as valid alternatives to the paramagnetic salts used in adiabatic demagnetization refrigerators (ADRs).^4,5^ In particular, the regular and dense organization of paramagnetic Gd(III) nodes in metal–organic frameworks (MOFs) has made possible the design of materials with a maximized density of spins, resulting in volumetric MCE values that compete or even outperform those of gadolinium gallium garnet (GGG),^5^ the reference material for magnetic cooling in the 20 to 0.5 K range.^6^ One advantage put forward for molecular-based coolers is that solution-based methods could be used for making films or localized deposits.^7^ This is relevant for applications where local cooling is required or deemed more efficient than bulk refrigeration. Indeed, ADRs and most cryogenic refrigeration setups are bulky, mismatching the devices to be cooled and limiting widespread applications.^8^ This has motivated intense research toward on-chip cooling, but the different solid-state methods developed thus far focus on the later stages of cooling, namely, < 500 mK or even lower temperatures.^9^ Although providing absolute Δ*T* only up to few hundreds mK, the *T*_i_/*T*_f_ ratios using these schemes can be quite large, especially when reaching the sub-mK range.^9d,9e^

**Figure 1:**
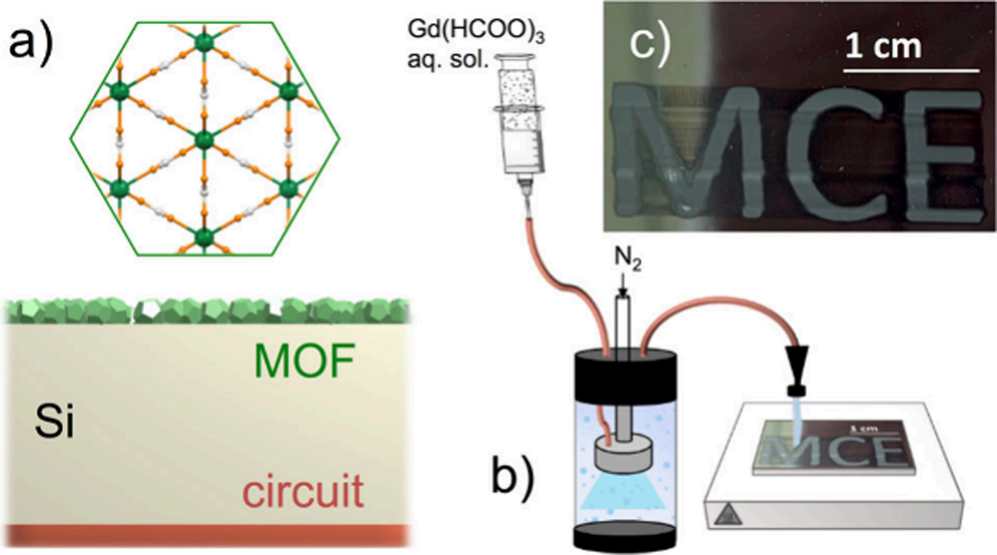
(a) Gd(HCOO)_3_ structure along its *c* axis and scheme of a locally cooled device. (b) Scheme of AJP setup. (c) SEM image of letters “MCE” written by AJP of Gd(HCOO)_3_ on Si.

Thin films of the best molecular-based coolers could provide efficient local magnetic refrigeration in the 10 K to tens of mK range, thanks to their very large volumetric MCE. Local cooling also would largely minimize the negative effect of the relatively poor thermal conductivity expected for molecular materials or MOFs.^10^ Although the deposition, growth, and patterning of porous MOFs have been the subject of intense research^11^ because of their relevance in many applications such as gas separation, storage, sensors, or catalysis, similar studies on dense MOFs are very rare. We have previously reported the growth of the gadolinium formate MOF Gd(HCOO)_3_ on a silicon surface modified with a monolayer bearing carboxylic acid functional groups.^12^ Although successful, only deposits of crystallites <30 nm thick were possible with this method, thus providing limited cooling power, with the additional drawback of the insulating organic layer. To form films of a dense MOF with high MCE and with controlled thickness on unmodified substrates, we turned to an emerging technique called aerosol jet printing (AJP, Figure 1). AJP is a contactless direct-write technique that uses a focused aerosol stream and can be used to directly pattern materials on virtually any substrate.^13^ While typically used to print inorganic materials as electronic components, some of us recently reported the first application of this technique to deposit a MOF, the ultramicroporous calcium squarate UTSA-280, with control over the deposit thickness.^14^ We report here the first application of this technique for a dense MOF, gadolinium formate, to obtain homogeneous films with thickness control. The MCE of the resulting deposits are determined through magnetization and heat capacity measurements, demonstrating a 50-fold improvement over previous films′ MCE performance.^12^ Moreover, cooling of the substrate by the deposit is directly measured for the first time.

## Experimental Methods

### Aerosol Jet Printing of Gd(HCOO)_3_ Films

AJP is a contactless-write technique which is based on an aerosol stream. A functional ink (solution) is aerosolized and carried to the substrate by a carrier gas (N_2_), as shown in Figure 1 and Scheme S1a. The viscosity of the solution is not as important as in other similar techniques (for example, inkjet printing). Setup used is the same as used before for the deposition of UTSA-280 coatings.^14^ Gd(HCOO)_3_ solutions of concentrations 0.5, 5, 10, and 20 mg/mL, i.e. respectively 0.0023, 0.0235, 0.0471, and 0.0942 mM, were prepared by ultrasonication of Gd(HCOO)_3_ powder in milli-Q water in an ultrasonic bath for 30 min. For AJP, the used solution is placed in a syringe, controlling the flux by a syringe pump. It is fed into a pneumatic atomizer (BLAM, CH Technologies) containing a laser-cut ruby orifice. Thanks to the atomizer and to the carrier gas, the liquid is broken into micrometer-sized droplets. The aerosol is carried by the gas stream to the deposition nozzle, which is the last part of the deposition setup. The nozzle allows droplets acceleration to the final substrate by a continuous jet. The nozzle is attached to a X-Y stage (modified PRUSA i3MK3). Its movement and the substrate bed temperature are programmed by GCode commands. The flux of the pump, speed of the writing and distance between lines were fixed at respectively 50 μL/min, 50 cm/min and 25 μm. The stage and therefore substrate temperature is either RT (ca. 22 °C) or 50 °C.

### Magnetic Measurements

Magnetic measurements were done with a Quantum Design MPMS XL magnetometer hosted by the Servicio de apoyo a la Investigación – SAI Universidad de Zaragoza. Magnetization vs temperature (*M* vs *T*, from 2 to 30 K, at 0.1 or 0.5 T) and Magnetization vs Field (*M* vs *B*, from 0 to 5 T, at 2–10 K) measurements were done, for both pristine 200 μm thick Si and 200 μm thick Si coated with various AJP deposits. The majority of measurements were performed with samples of 0.3 cm^2^. The 5 × 6 mm^2^ rectangular pieces of Si were held vertically within the standard plastic straw typically used with this commercial magnetometer. The magnetic field was therefore applied parallel to the deposit surface.

### Heat Capacity and MCE Direct Measurements

Heat capacity and MCE direct measurements were made with the ^3^He heat capacity option of a Quantum Design 9 T Physical Properties Measurement System hosted by the Servicio de apoyo a la Investigación – SAI Universidad de Zaragoza. All experiments were done on 0.0625 cm^2^ pieces of 200 μm thick Si, either pristine or coated with various AJP deposits. The sample was fixed to the sapphire sample holder with little Apiezon N grease (see Scheme S2). Heat capacity measurements were made down to 0.35 K in zero-field and at 1 T, 3 and 5 T applied magnetic field. These measurements are done under high vacuum. Direct measurements of MCE were performed with the same setup by following the resistance of a Cernox (CX-1010) resistance thermometer attached to the bottom side of the sapphire sample holder (see Scheme S2) upon applying and removing magnetic fields at 100 Oe/s. The thermometer resistivity data are corrected for magnetoresistive effects measured experimentally with a bare Si substrate. The corrected thermometer resistivities are then transformed into temperatures through the thermometer calibration (see Figure S13).

## Results and Discussion

**Figure 2:**
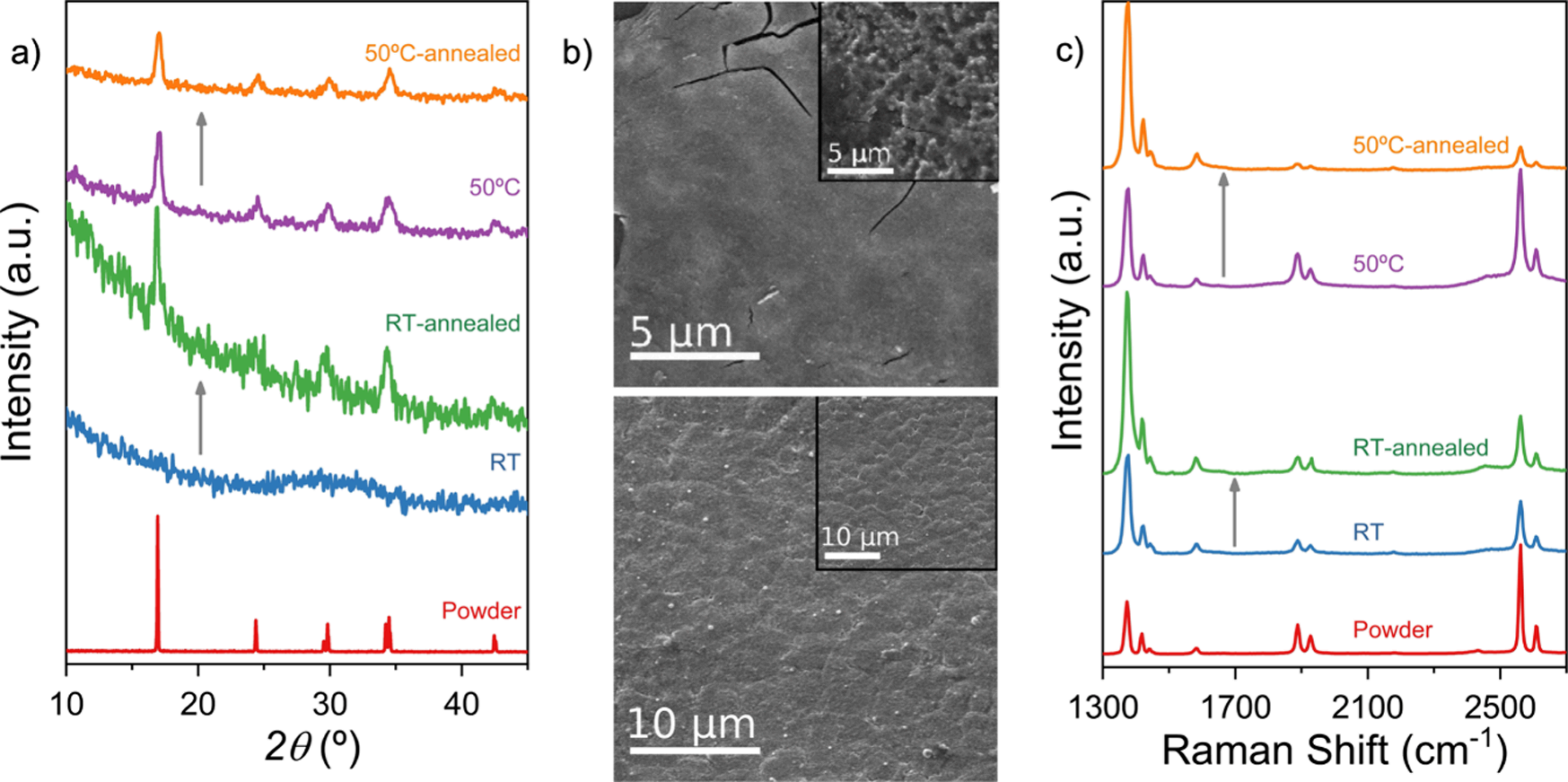
Characterization of AJP films. (a) GIXRD characterization of RT (20 passes) and 50 °C (3 passes) depositions on Si by AJP, as initially obtained and after annealing at 80 °C, compared with the bulk powder. (b) Frontal SEM images of 1 pass AJP deposits made on the Si substrate at RT (top) and 50 °C (bottom). Inset: Frontal SEM images of the same deposits after annealing at 80 °C. (c) Raman spectra of AJP deposits on Si, as initially obtained and after annealing at 80 °C, compared with the bulk powder spectrum.

Among the Gd-based MOFs with very large MCE,^5^ we selected Gd(HCOO)_3_ because it remains one the few materials surpassing GGG at relatively low fields,^5c^ and due to its high solubility in H_2_O. This is essential because the AJP film formation technique requires the material’s precursors to be in solution(s). Also, Gd(HCOO)_3_ crystallizes from concentrated aqueous solutions containing formic acid and Gd(III) ions resulting from the acid hydrolysis of Gd_2_O_3_. We have focused on silicon as the substrate because of its wide use in device fabrication and its low heat capacity in the temperature range of interest. Pieces of Si(100) wafers of various thicknesses were cleaned with piranha solution and placed on the sample stage of the AJP setup that had previously been used for deposition of the porous MOF UTSA-280 (see Supporting Information).^14^ Continuous films of Gd(HCOO)_3_ were successfully obtained by AJP using a 10 mg/mL Milli-Q water solution of Gd(HCOO)_3_, fixing the flow rate at 50 μL/min, the linear speed of the nozzle at 50 cm/min, and the distance between lines at 25 μm. The effect of the substrate temperature was studied by making films on a substrate either at room temperature (RT, ca. 22 °C) or at 50 °C. Whereas the RT films are amorphous and present a glass-like continuous surface, the films prepared with the substrate at 50 °C are homogeneous and polycrystalline, and the GIXRD patterns fully coincide with those of the bulk material (Figure 2a and b). Raman spectra in both cases are identical with that of the bulk material (Figure 2c), indicating that the material deposited at RT is also Gd(HCOO)_3_, albeit not crystalline.

The magneto-thermal properties of the deposits also support the successful deposition of Gd(HCOO)_3_. After proper scaling, the isothermal magnetization *M* versus magnetic field *B* data at 2 K are close to the Brillouin function for a Gd(III) ion with g = 2.02 and *S* = 7/2 (Figure S1), as expected for a paramagnet, and virtually identical with those of the bulk material. Similarly, the temperature dependence of the scaled magnetic susceptibility (χ) of the deposits, determined in the 2–30 K range, follows the same Curie–Weiss law as that of the bulk material (Figure S2). The scaling factors allow indirect determination of the mass of Gd(HCOO)_3_ deposited, and thereby evaluation of the efficiency of the deposition process upon repeated AJP passes (see below). The temperature dependence of the zero-field heat capacity of the deposits is also informative: the peak at 0.8 K characteristic of the magnetic order exhibited by the bulk material^5c^ is observed in the case of the crystalline films obtained with the substrate at 50 °C, but is absent for the amorphous films formed on the substrate at RT (Figure S3). Clearly, the lack of crystalline order impedes or significantly weakens the long-range magnetic order. Notably, the occurrence of magnetic order sets a temperature lower-bound for the cryogenic refrigeration by magnetocaloric materials because their entropy drops drastically at the order transition. The formation of an amorphous film could therefore represent an advantage if the amorphous material retains similar MCE properties to those of the crystalline phase, possibly allowing reaching lower temperatures.

A range of concentrations of the Gd(HCOO)_3_ solution were studied for optimization. At 20 mg/mL, the solution is too close to saturation, resulting in instabilities during the AJP deposition process. This is undesirable as the high concentration may favor partial nozzle obstruction. Lower concentrations of 0.5 and 5 mg/mL, however, did not provide satisfactory deposition conditions either. At 0.5 mg/mL, effectively no material deposited on the silicon substrate, probably due to too low local surface concentration of the material components. Using a 5 mg/mL solution, the first AJP pass does not result in continuous coverage of the substrate, which only progressively improves when more passes are performed (Figure S4). Here too, this result likely indicates that the formation and/or crystallization of Gd(HCOO)_3_ requires a minimum local surface concentration. Interestingly, when using an optimal concentration of 10 mg/mL, the substrate is fully covered even after 1 pass. This contrasts with UTSA-280, where full coverage was not reached even after 10 passes.^14^ In addition, the Gd(HCOO)_3_ crystallites formed when the substrate is at 50 °C are much smaller and isotropic compared with the relatively long needles formed by UTSA-280. These differences may in part result from the hydrophilic nature of Gd(HCOO)_3_, which allows efficient coverage of the substrate surface by the aerosol droplets, thus permitting concomitant nucleation and growth processes over the entire surface covered by the droplet. This seems to point at a relatively efficient deposition process, which we assessed indirectly through the magnetic properties of the deposits, which allow to estimate their masses (Figure S5 and Table S1). The estimated masses are on average ca. 1.8% of the expected masses based on the concentration and flow of the injected solution, the printing process duration, and the printed surface. Considering an atomization efficiency of 10%, the deposition efficiency for Gd(HCOO)_3_ is ca. 18%, approximately twice that estimated for UTSA-280.^14^

Thermal annealing of the deposits was performed to evaluate its potential effect on the density and crystallinity of the films. Deposits were heated by placing the substrate directly on a hot plate at 80 °C, for 3 h under ambient conditions, and then let cool to RT. GIXRD measurements show that the amorphous deposits originally formed with the substrate at RT have crystallized upon annealing, with the patterns corresponding to those of the bulk material (Figure 2a). This indirectly confirms that the original amorphous material is indeed Gd(HCOO)_3_. SEM images also exhibit the effect of this crystallization, the original continuous glass-like appearance having changed to a rougher polycrystalline topography (Figure 2b). The IR spectrum of the annealed deposit also shows sharper O−C−O and C−H bands, similar to those of the bulk material (Figure S6). In contrast, annealing of AJP films formed with the substrate at 50 °C does not result in any significant modifications. The peak intensity and width of the GIXRD patterns remain unaltered (Figure S7). The topography of the deposit is also unchanged, although thicker films show some unwanted cracking. Altogether, thermal annealing allows crystallization of the initially amorphous deposits formed at RT, but is not useful if the AJP process is performed at 50 °C.

**Figure 3:**
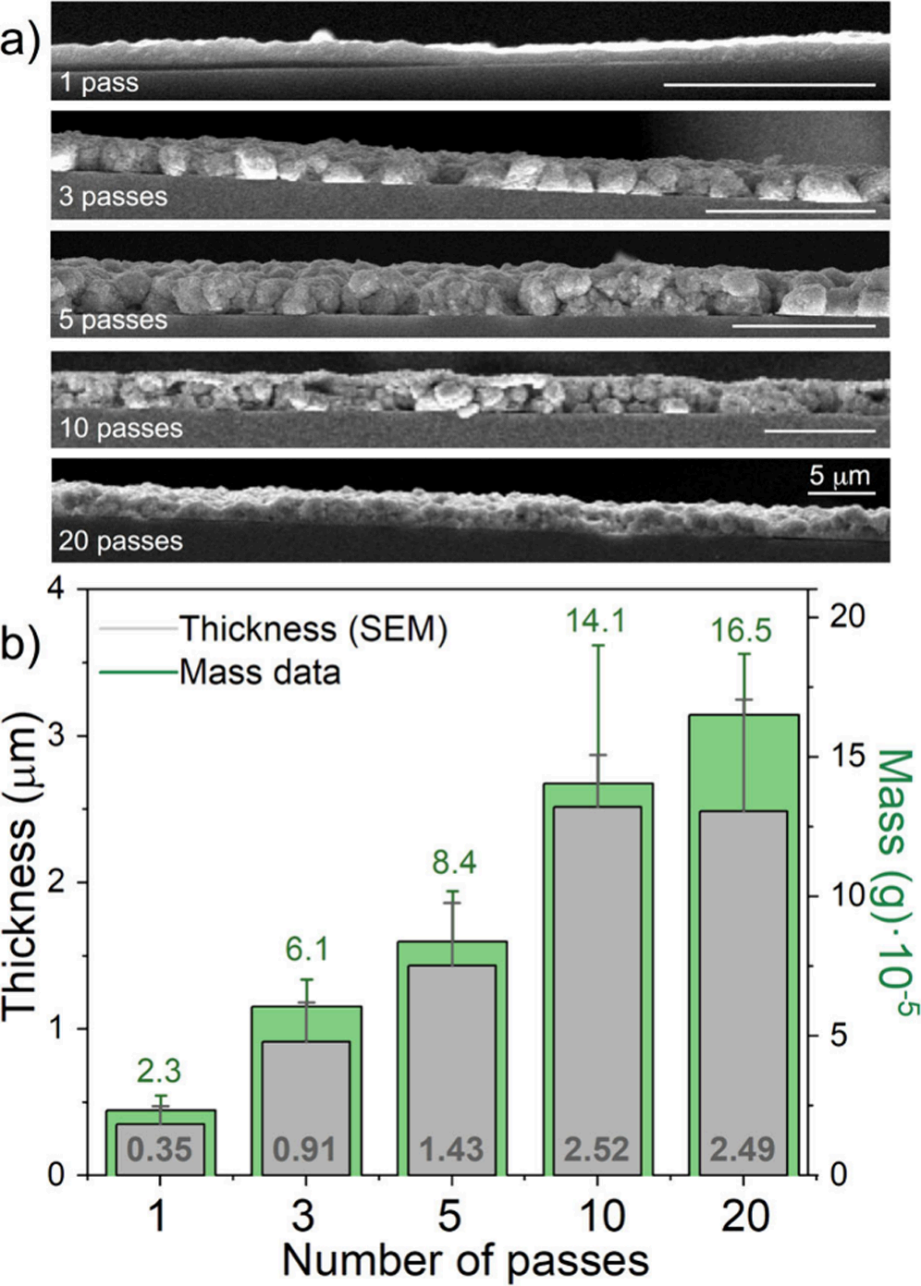
(a) Transverse SEM images of films obtained with 1, 3, 5, 10, and 20 AJP passes. The size bar is 5 μm in all images. (b) Thickness of deposits formed with 1 to 20 AJP passes derived from SEM images, and mass of the deposits as derived from *M* vs *B* data. Error bars correspond to the deviation of, at least, 2 samples.

Having determined the conditions for homogeneous, continuous, and crystalline deposition of the high MCE Gd(HCOO)_3_ MOF, the next goal was increasing the thickness of the deposits in a controlled way to provide an adjustable cooling power function of the device to be refrigerated. Because using higher concentrations of the starting solution proved challenging, we took advantage of the automated nature of the AJP technique and made deposits on Si substrates at 50 °C at the optimal concentration of 10 mg/mL with 1, 3, 5, 10, and 20 passes. Transverse SEM images (Figure 3a) show the increased, homogeneous thickness of the film. The thickness increase is perfectly linear from 1 to 5 passes (from 0.35 to 1.43 μm), but from then on the deposition process loses efficiency (Figures 3b and S8). The 2.52 μm thickness observed for the 10 passes deposit is only slightly inferior to that expected for a regular linear increase, but the 20 passes deposit does not result in a significantly larger amount of deposited material. This is likely due to the relatively high concentration of the solution that eventually leads to partial obstruction of the nozzle. Although cleaning of the nozzle at an intermediate stage did not significantly improve the final thickness, we are confident that improvements to our lab-made AJP setup should allow reaching a high number of passes without loss of efficiency. The mass of the deposits determined indirectly by scaling the *M* vs *B* and *χ* vs *T* data (Figures S2 and S5, Table S1) exhibit a very similar trend to that obtained from electron microscopy. Altogether, AJP allows a fine control over the thickness of Gd(HCOO)_3_ films from 0.35 to 2.52 μm, which could probably be expanded to higher thicknesses.

**Figure 4:**
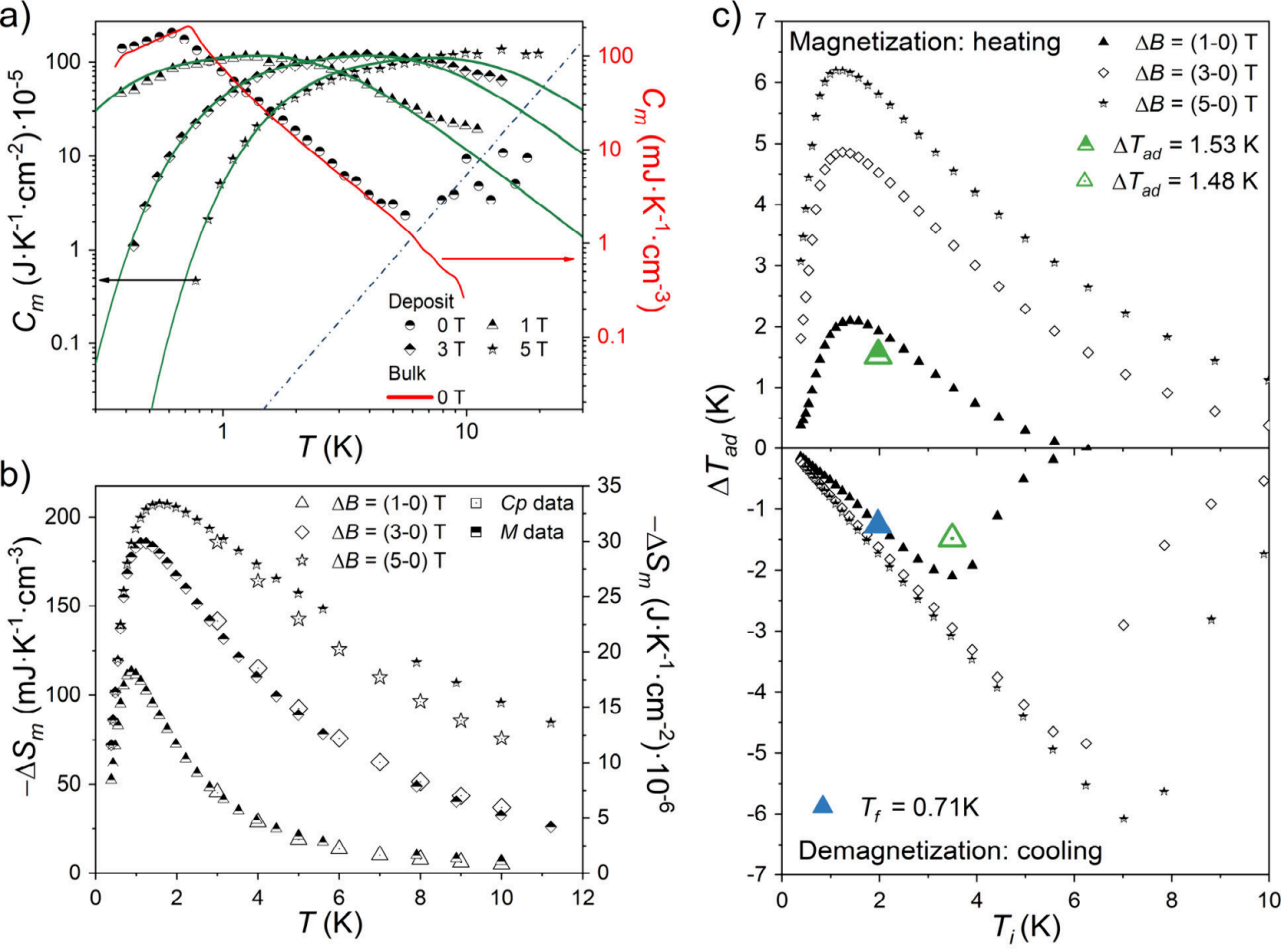
(a) Temperature dependence of *C*_m_ for different applied fields *B* for a 20 passes deposit. The zero-field data for bulk Gd(HCOO)_3_ is shown for comparison. Solid green lines are calculated Schottky contributions for 1, 3, and 5 T. The blue dashed line is the lattice contribution of the Gd(HCOO)_3_ calculated for a Debye temperature θ_D_ = 168 K.^5c^ (b) Temperature dependence of Δ*S*_m_ for different Δ*B*, derived from magnetic heat capacity and magnetization data. (c) Temperature dependence of Δ*T*_ad_ of the whole sample {20 passes deposit + Si} derived from the total entropy *S*(*T*) for different field changes Δ*B*. The *x* axis represents the starting temperature of the adiabatic process. Larger colored symbols correspond to the Δ*T*_ad_ determined through direct measurements (see Figures 5a and S13).

To evaluate the MCE properties of the deposits, isothermal magnetization data from 2 to 10 K were determined for deposits formed with the substrate at 50 °C and higher amounts of MOF material, that is, deposits obtained with 5, 10, and 20 AJP passes (Figure S9). The magnetic entropy changes Δ*S*_m_(*T*, Δ*B*) for different applied field changes Δ*B* = *B*_f_ – *B*_i_ can be indirectly derived from these data using the Maxwell relation, i.e.  Expressed per unit of surface area, −Δ*S*_m_ at Δ*B* = 3 T and 3 K reaches values of 12.5 × 10^–6^ J·K^–1^·cm^–2^ for a 5 passes deposit and 30.5 × 10^–6^ J·K^–1^·cm^–2^ for both 10 and 20 passes deposits. These values are more than 50 times higher than those estimated in the only previous report on magnetocaloric MOF films,^12^ which is in line with the much larger thicknesses obtained here. The derived Δ*S*_m_ are altogether in excellent agreement with those reported for the bulk material (Figure S10),^5c^ the −Δ*S*_m_ mentioned above for Δ*B* = 3 T and 3 K corresponding to a volumetric −Δ*S*_m_ of 140.4 mJ·K^–1^·cm^–3^. In addition, low temperature heat capacity measurements were done on a 20 passes deposit at several applied magnetic fields to indirectly determine both −Δ*S*_m_ and Δ*T*_ad_. The magnetic component of the heat capacity *C*_m_ was calculated by subtracting the lattice contribution previously determined for the bulk material,^5c^ as well as the heat capacity of the Si, which was determined experimentally under the same experimental conditions. Figure 4a shows the excellent agreement between the data for the scaled deposit and those of the bulk material. As for the bulk material, application of a 1 T field is sufficient to overcome the magnetic order, and *C*_m_ values agree well with Schottky contributions (green solid lines), at all applied fields. The scale factor again allows an estimation of the mass of Gd(HCOO)_3_ deposited, which is in good agreement with that derived from magnetization and susceptibility data (Table S1). The magnetic entropy *S*_m_ is determined by integration, i.e. , giving the maximum entropy expected *S*_*m*_^max^ = *R* ln(2*S* + 1) = 17.29 J·mol^–1^·K^–1^ = 59.15 J·kg^–1^·K^–1^ per Gd(III), where *S* = 7/2 and *R* is the gas constant (Figure S11). The magnitude defining the MCE, Δ*S*_m_, is then obtained numerically from the *S*_m_(*T*) curves for different field changes Δ*B* (Figures 4b and S12). An excellent agreement is found for the change in magnetic entropy Δ*S*_m_ of the deposits as determined through calorimetric and magnetic data for the lower 1 and 3 T fields (Figure 4b). These are also similar to those of the bulk material (Figure S12), altogether confirming that the obtained deposits maintain the material’s cooling capacity.

**Figure 5:**
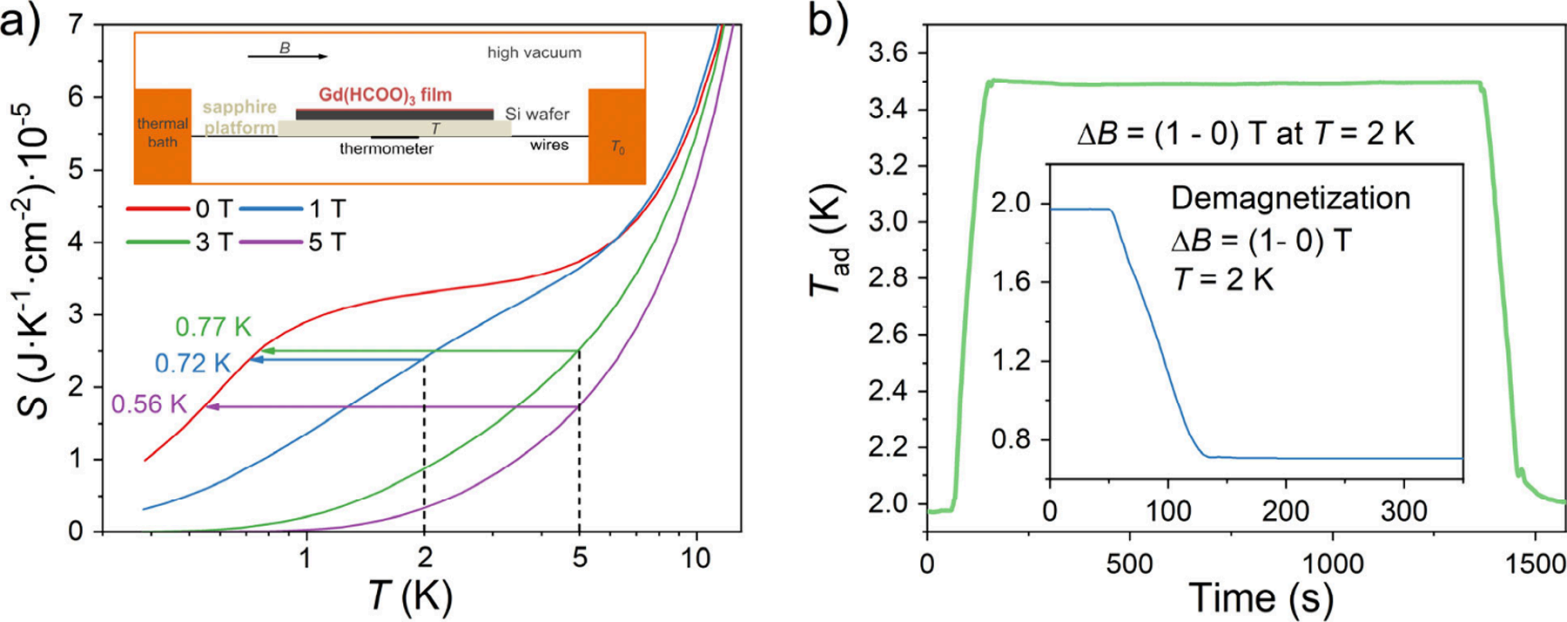
(a) Temperature dependence of total entropy *S*(*T*) at different fields (0, 1, 3, and 5 T) for a Si wafer with a 20 passes Gd(HCOO)_3_ deposit, as obtained by integration of the total heat capacity, i.e., *S* = ∫_0_^*T*^*C*/*T* d*T*. Arrows show the cooling produced by adiabatic demagnetization processes starting from either 2 or 5 K. Inset: scheme of the experimental set-up used for heat capacity and direct measurements of MCE. (b) Time dependence of the inferred *T*_ad_ of the whole system {deposit + Si + sapphire holder + thermometer} (green line) for a full magnetization-demagnetization cycle at 100 Oe/s sweep rate with the thermal bath at 2 K and *B* = 1 T. Inset: Inferred cooling process upon adiabatic demagnetization from *B* = 1 T and *T*_0_ = 2 K.

To determine the cooling capacity of our deposits, we then considered the total entropy *S*(*T*) data at the different applied fields derived by integration of the total heat capacity (Figure 5a). These allow indirect determination of the heating and cooling resulting from an adiabatic magnetization (heating, Figure 4c, top) and demagnetization (cooling, Figure 4c, bottom), respectively, for the whole sample {deposit + Si}. A 20 passes deposit should thus be able to cool itself and the 200 μm Si substrate from 6.2 to 1.4 K by removing a 3 T magnetic field. Using a relatively low magnetic field of 1 T, the deposit would cool its substrate from 2 K to below 1 K in a single adiabatic demagnetization step, while cooling from 5 K to below 1 K is also possible in a single demagnetization step using higher fields of 3 or 5 T, as shown with arrows in Figure 4d. A single demagnetization at 2 K would cool the whole sample {deposit + Si} to below 1 K in all cases. The estimated cooling from 2 to 0.72 K produced by removing a magnetic field of 1 T is most likely the optimal, as the final temperature is close to the ordering temperature of the magnetocaloric material used.

Eventually, we also determined the cooling capacity of our deposit through direct measurements. As previously reported for bulk macroscopic samples,^5c,15^ this was done by continuously recording the temperature of the whole system {deposit + Si + sapphire platform + thermometer} (see inset in Figure 5a and Scheme S2) while applying and removing a magnetic field, letting the system relax to the bath temperature after each step. A first step involves the correction of magnetoresistive effects determined by performing the same process for an uncovered piece of Si (see Figure S13). Because of the lack of adiabaticity, the as-measured temperature variations are relatively small, and estimated values of *T*_ad_ are obtained numerically from the measured *T*, by estimating the entropy losses/gains Δ*S* to/from the thermal bath, calculated as κ(*T* – *T*_bath_) using the known wires thermal conductance κ(*T*), and considering that , where *C* is the as-measured total heat capacity for the whole system {deposit+Si+sapphire platform} (see Figures S13 and S14). Figure 5b shows a full magnetization–demagnetization cycle at 2 K and 1 T. Both the derived adiabatic heating to ca. 3.5 K and cooling back to ca. 2.1 K upon respectively applying and removing the magnetic field are in good agreement with the indirect estimation from calorimetric data, as shown in Figure 4c. The slightly smaller Δ*T*_ad_ values obtained through direct measurements are likely due to the necessity to additionally warm/cool the sapphire sample platform and the thermometer, which is not considered in indirect determination as the entropy used is that of the whole sample {deposit + Si}. Importantly, the final *T*_ad_ at the end of the full magnetization–demagnetization cycle is very close to the starting temperature, thereby validating the corrections made. Using the same data, albeit starting with the system at 2 K in an applied magnetic field of 1 T, adiabatic demagnetization results in the cooling of the whole system down to 0.72 K. Again, the obtained value is in excellent agreement with that derived indirectly from *S*(*T*).

## Conclusions

In conclusion, we have shown that homogeneous and crystalline deposits of the MOF Gd(HCOO)_3_ can be formed from aqueous solutions on unmodified silicon substrates using the AJP technique. Repeated deposition cycles allow control of the deposit thickness up to at least 2.5 μm. The deposits maintain the very high magnetocaloric performance of the bulk material, resulting in an unprecedently high surface cooling capacity as measured through the indirect determination of the change in *S*_m_ and *T*_ad_ of the whole sample {deposit + Si substrate} upon removal of an applied magnetic field. Direct measurements of the temperature variation demonstrate this cooling capacity, as the time dependence of the inferred *T*_ad_ shows the deposit should be able to cool itself, a 200 μm thick Si wafer, and a sapphire platform from 2 K to <1 K by removing a relatively small magnetic field of 1 T in adiabatic conditions. Overall, our work demonstrates the potential of thin films of metal–organic magnetic coolers for local on-chip magnetic refrigeration to sub-K temperatures. We envision that the method should be applicable to other molecular-based coolers with lower ordering temperatures,^4b,4c,4f^ thereby allowing cooling to the mK range. The application of the method to materials useful in different temperature ranges could also allow the fabrication of multimaterial/multistage cooling films. Alternatively, deposition of films with preferential orientation of an anisotropic magnetocaloric material would open the possibility of local refrigeration by rotation of a device.^16^

## Supporting Information Available

Additional experimental details, additional magneto-thermal data, IR spectra, additional SEM images, details of indirect determination of the magnetocaloric effect and of its direct measurements (PDF)
Aerosol jet printing process movie (MOV)

## Supplementary Material

cm4c00909_si_001.pdf

cm4c00909_si_002.mov
