# Supplementary material for: Gd Metal–Organic Framework Thin Film for On-Chip Local Magnetic Refrigeration
Source: Chem Mater. 2024 Aug 20;36(17):8239–46. doi: 10.1021/acs.chemmater.4c00909 (PMC11404032; doi:10.1021/acs.chemmater.4c00909)
Supplement: Supplementary file 1 — cm4c00909_si_001.pdf [file cm4c00909_si_001.pdf]

Supporting information for the manuscript

Gd metal-organic framework thin film for on-chip local magnetic refrigeration

Inés Tejedor,[1] Dmitry E. Kravchenko,[2] Jesús Gandara-Loe,[2] Rob Ameloot,[2,*] Ignacio Gascón[1] and Olivier Roubeau[1,*]

[1]Instituto de Nanociencia y Materiales de Aragón (INMA), CSIC and Universidad de Zaragoza, Zaragoza 50009 ,Spain
[2]Centre for Membrane Separation, Adsorption, Catalysis and Spectroscopy, KU Leuven, Celestijnenlaan 200F, Leuven 3001, Belgium
*rob.ameloot@kuleuven.be, roubeau@unizar.es

Table of contents

Experimental details p. S2

Figure S1. Isothermal magnetization data at 2 K for a 1 AJP pass deposit prepared at 50ºC p. S5

Figure S2. Temperature dependence of χ, χ^{-1} and χT for 1-20 AJP passes deposits p. S6

Figure S3. Temperature dependence of C_m in zero field for RT and 50ºC AJP deposits p. S7

Figure S4. Cross section SEM images of deposits obtained at 5 mg/mL water concentration p. S8

Figure S5. Surface *M vs. B* at 2 K for 1-20 passes deposits and calculated mass deposited p. S9

Table S1. Mass of deposit per area as derived from magnetic and heat capacity data p. S10

Figure S6. IR spectra and zoom of Raman spectra of AJP deposits prepared at RT and 50ºC, and after annealing at 80ºC p. S11

Figure S7. GIXRD patterns for a 10 passes AJP prepared at 50ºC upon annealing to 80ºC p. S12

Figure S8. Deposit thickness *vs.* number of AJP passes derived from SEM images p. S13

Figure S9. Isothermal field dependences at 2-10 K for a 5 AJP passes deposit p. S14

Figure S10. ΔS_m *vs. T* of 5, 10 and 20 AJP passes deposits from *M vs. B* data p. S15

Figure S11. S_m *vs. T* at various *B* for a 20 AJP passes deposit from calorimetric data p. S16

Figure S12. ΔS_m and ΔT_{ad} *vs. T* of a 20 AJP passes deposit for various ΔB p. S17

Figure S13. Direct measurements of the magnetic heating/cooling by a 20 passes deposit p. S18

Figure S14. Time evolution of T_{ad} showing the successive steps in its numerical estimation p. S19

Experimental details

Reagents

HCOOH (ChemLab CAS:64-18-6), Gadolinium (III) oxide (nanopowder, 99.99+% (REO), Thermo Scientific Chemicals, CAS: 12064-62-9), Sufuric acid (95-97%, Supelco, CAS:7664-93-9) and Hydrogen peroxide (35%, ChemLab. 7722-84-1). Ultra-pure milli Q water, resistivity 18.2 MΩ·cm

Synthesis of $Gd(HCOO)_3$

Bulk crystalline powder of $Gd(HCOO)_3$ was obtained by reaction of vapours of HCOOH with Gd_2O_3 in a closed vessel at 80ºC during 24 hours in stoichiometric conditions. Purity was checked by powder X-ray diffraction and IR and Raman spectroscopies.

Substrates

Si wafers oriented along <100> axis were used. Si thickness were 200, 325 and 725 μm. All were cleaned by a piranha solution. Magneto-thermal measurements were done on samples prepared on 200 μm Si wafers.
CAUTION: piranha solutions are extremely corrosive to organic substances and may irritate the respiratory tract if vapor is inadvertently inhaled. Piranha solutions are extremely energetic and may result in explosion or injury if not handled with extreme caution.

AJP solution preparation

Solutions were prepared by ultrasonication of $Gd(HCOO)_3$ powder in milli-Q water in an ultrasonic bath for 30 minutes. Concentrations used were 0.5, 5, 10 and 20 mg/mL, *i.e.* respectively 0.0023, 0.0235, 0.0471 and 0.0942 mM.

Aerosol Jet Printing (AJP) Setup

AJP is a contactless-write technique which is based on an aerosol stream. A functional ink (solution) is aerosolized and carried to the substrate by a carrier gas (N_2), as shown in Scheme S1a. The viscosity of the solution is not as important as in other similar techniques (for example, inkjet printing).

Setup used is shown in Scheme 1a. It is the same setup used before for the deposition of UTSA-280 coatings.[1] $Gd(HCOO)_3$ milliQ water solution is placed in a syringe, controlling the flux by a syringe pump. It is fed into a pneumatic atomizer (BLAM, CH Technologies) containing a laser-cut ruby orifice. Thanks to the atomizer and to the carrier gas, the liquid is broken into micrometer-sized droplets. The aerosol is carried by the gas stream to the deposition nozzle, which is the last part of the deposition

[1] Kravchenko, D. E.; Matavz, A.; Rubio-Giménez, V.; Vanduffel, H.; Verstreken, M.; Ameloot, R. Aerosol Jet Printing of the Ultramicroporous Calcium Squarate Metal-Organic Framework. *Chem. Mater.* **2022**, *34*, 6809-6814

setup. The nozzle allows droplets acceleration to the final substrate by a continuous jet. The nozzle is attached to a X-Y stage (modified PRUSA i3 MK3). Its movement and the substrate bed temperature are programmed by GCode commands. Some conditions are fixed: flux of the pump (50 µL/min), speed of the writing (50 cm/min), distance between lines (25 µm). In our studies, the stage and therefore substrate temperature is either RT (ca. 22ºC) or 50ºC. Si substrates of various sizes were coated by moving the nozzle in the pattern shown in Scheme S1b.

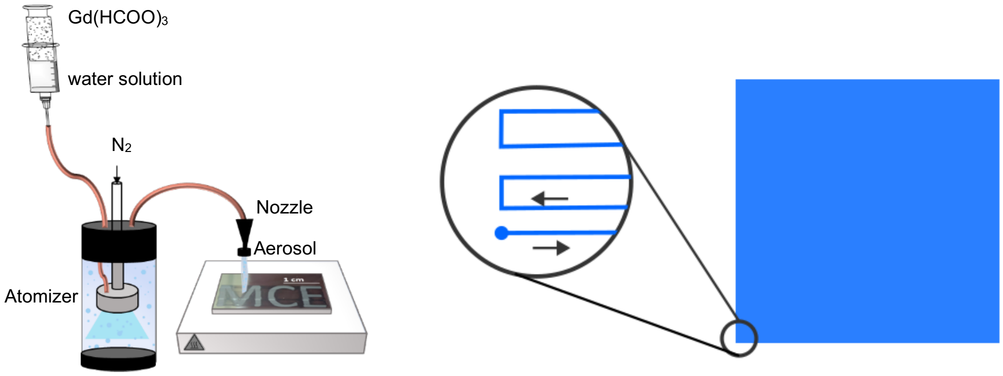

Scheme S1. a) AJP setup. b) pattern of the movement of the nozzle

Scanning electron microscopy (SEM)

Images were recorded by using either a Philips/FEI XL-30 FEG instrument (KU Leuven) or an INSPECT-F50 instrument (LMA, INMA, University of Zaragoza), both operating at an accelerating voltage of 10 keV. Samples were previously sputter-coated with 2 nm of Pt.

GIXRD measurements

Grazing-incidence X-ray diffraction (GIXRD) patterns were recorded in reflection mode over a 4-50° 2θ range with an incident beam angle of 0.2°. A PIXcel3D solid state detector and a Cu anode (with Cu Kα1 = 1.5406 Å and Cu Kα2 = 1.5444 Å wavelengths) were used for all measurements.

Infrared Spectroscopy

IR spectra were acquired with either a Varian 670 FT-IR spectrometer (KU Leuven) equipped with an MCT detector or a FTIR Bruker Vertex 70 spectrometer (INMA, University of Zaragoza).

Raman Spectroscopy

Raman spectra were obtained with a WITec Alpha 300 equipped with a confocal microscope (INMA, University of Zaragoza). The laser used was 532 nm. Measurements were made with 25 accumulations, 3 seconds of integration time and a voltage of 1.035 mV.

Magneto-thermal characterization

Magnetic measurements were done with a Quantum Design MPMS XL magnetometer hosted by the Servicio de apoyo a la Investigación – SAI Universidad de Zaragoza. Magnetization *vs.* temperature (*M vs.T*, from 2 to 30 K, at 0.1 or 0.5 T) and Magnetization *vs.* Field (*M vs. B*, from 0 to 5 T, at 2-10 K) measurements were done, for both pristine 200 µm thick Si and 200 µm thick Si coated with various AJP deposits. The majority of measurements were performed with samples of 0.3 cm^2. The 5×6 mm^2 rectangular pieces of Si were held vertically within the standard plastic straw typically used with this commercial magnetometer. The magnetic field was therefore applied parallel to the deposit surface.

Heat capacity and MCE direct measurements were made with the ^3He heat capacity option of a Quantum Design PPMS 9 T Physical Properties Measurement System hosted by the Servicio de apoyo a la Investigación – SAI Universidad de Zaragoza. All experiments were done on 0.0625 cm^2 pieces of 200 µm thick Si, either pristine or coated with various AJP deposits. The sample was fixed to the sapphire sample holder with little Apiezon N grease (see Scheme S2). Heat capacity measurements use the relaxation method[2] and were made down to 0.35 K in zero-field and at 1 T, 3 T and 5 T applied magnetic field. These measurements are done under high vacuum. Direct measurements of MCE were performed with the same set-up by following the resistance of a Cernox (CX-1010) resistance thermometer attached to the bottom side of the sapphire sample holder (see Scheme S2) upon applying and removing magnetic fields at 100 Oe/s. The thermometer resistivity data are corrected for magneto-resistive effects measured experimentally with a bare Si substrate. The corrected thermometer resistivities are then transformed into temperatures through the thermometer calibration (see Figure S13).

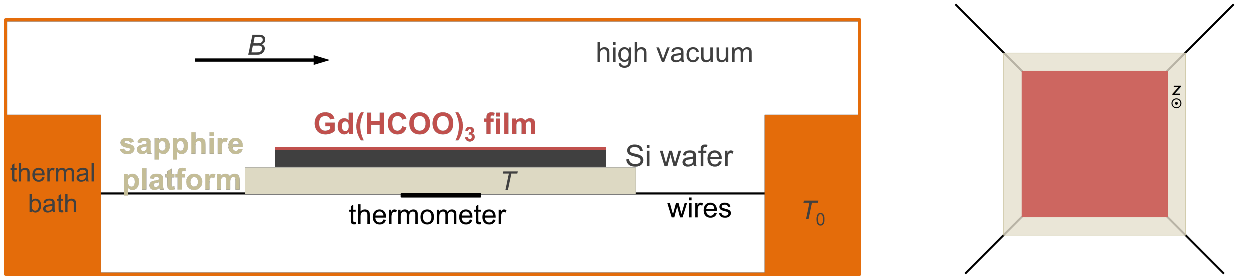

Scheme S2. Schematic side (left) and top (right) views of the set-up used for heat capacity and direct MCE measurements. The metallic wires thermal conductance is determined experimentally by measuring an oxygen-free copper block.

---

[2] a) see quantum Design application note at https://qd-uki.co.uk/wp-content/uploads/2019/07/Heat-Capacity-and-Helium-3-Application-Note.pdf, retrieved on 12/06/024; b) for the application of the two-tau model to determine heat capacity from relaxation data, see J. S. Hwang, K. Lin, C. Tien, *Rev. Sci. Instrum.* **1997**, 68, 94

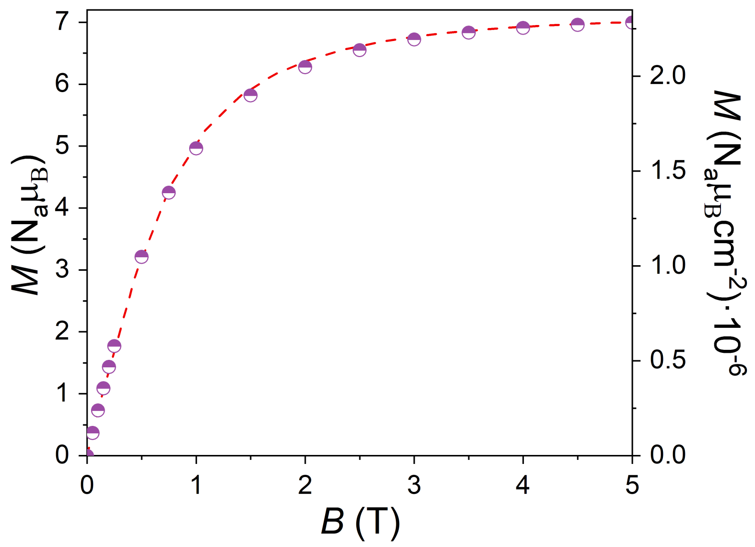

Figure S1. Magnetization *M* vs. magnetic field *B* data recorded at 2 K for a 1 pass sample prepared at 50 ºC (◓, mass calculated: $2.87 \cdot 10^{-2}$ mg). The raw data is obtained after subtraction of the diamagnetic component corresponding to the Si wafer, determined experimentally, and then scaled to reach the value calculated according to Brillouin function for an *S* = 7/2 spin and g = 2.02 at the highest field of 5 T. This gives the magnetization in Bohr magneton units $N_A\mu_B$. The whole data set agrees very well with the Brillouin function, shown as dashed red line. The corresponding surface magnetization expressed in $N_A\mu_B cm^{-2}$ is directly obtained considering the area of the Si wafer used (5×6 mm^2).

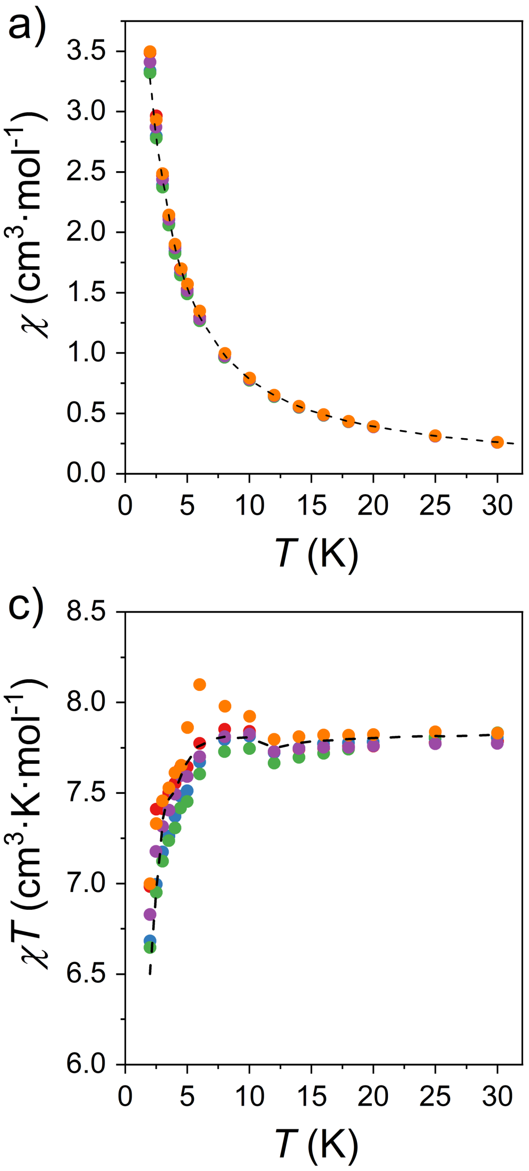

Figure S2. Temperature dependence of the magnetic susceptibility χ (a), χ^{-1} (b) and χT (c and d as semi-log plot) for deposits obtained with the Si substrate at 50ºC and increasing number of AJP passes: 1•, 3•, 5•, 10• and 20• passes. The data are corrected for the diamagnetic response of the Si substrate and scaled properly to the bulk powder data, which is shown as dashed black line for comparison. Note that the anomalies in the vicinity of *ca.* 4-5 K and *ca.* 11-12 K are associated with changes in the temperature control of the magnetometer.

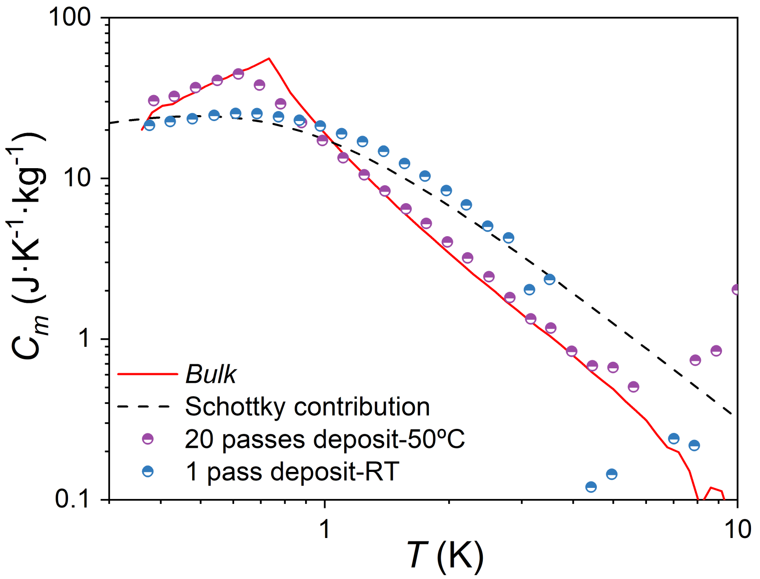

Figure S3. Temperature dependence of the magnetic component of the heat capacity C_m in zero field for AJP deposits prepared with the Si substrate at room temperature and 50ºC. Raw data are corrected of the heat capacity of the 2.5×2.5 mm^2 Si wafer and scaled adequately. The bulk material zero-field C_m is shown as a full red line for comparison.

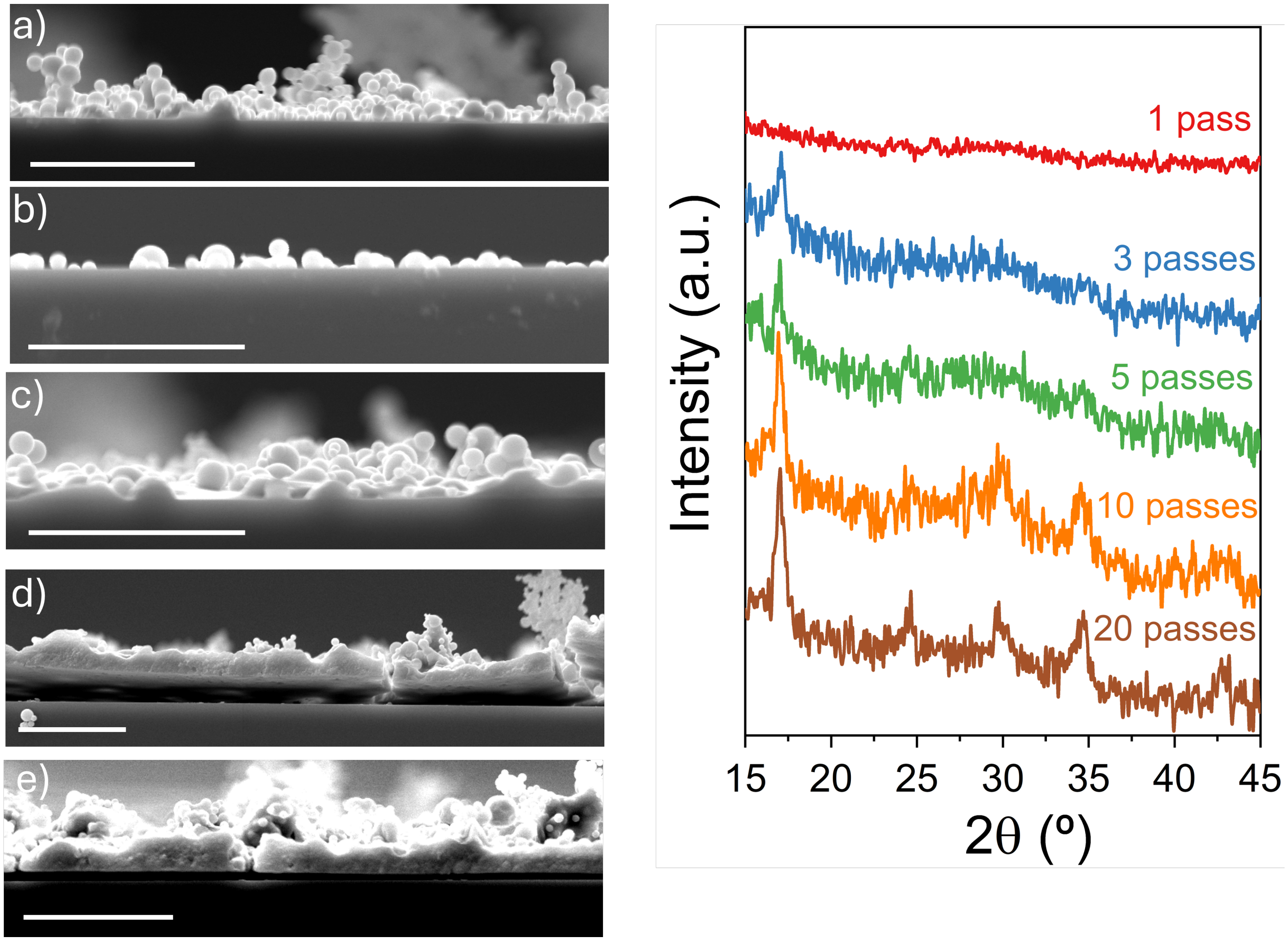

Figure S4. Left: SEM lateral cross section images of AJP deposits obtained using a 5 mg/mL water solution, and the Si substrate at 50ºC: a) 1 pass; b) 3 passes; c) 5 passes; d) 10 passes and e) 20 passes. In each image, the scale bar is 5 μm. Right: corresponding GIXRD patterns.

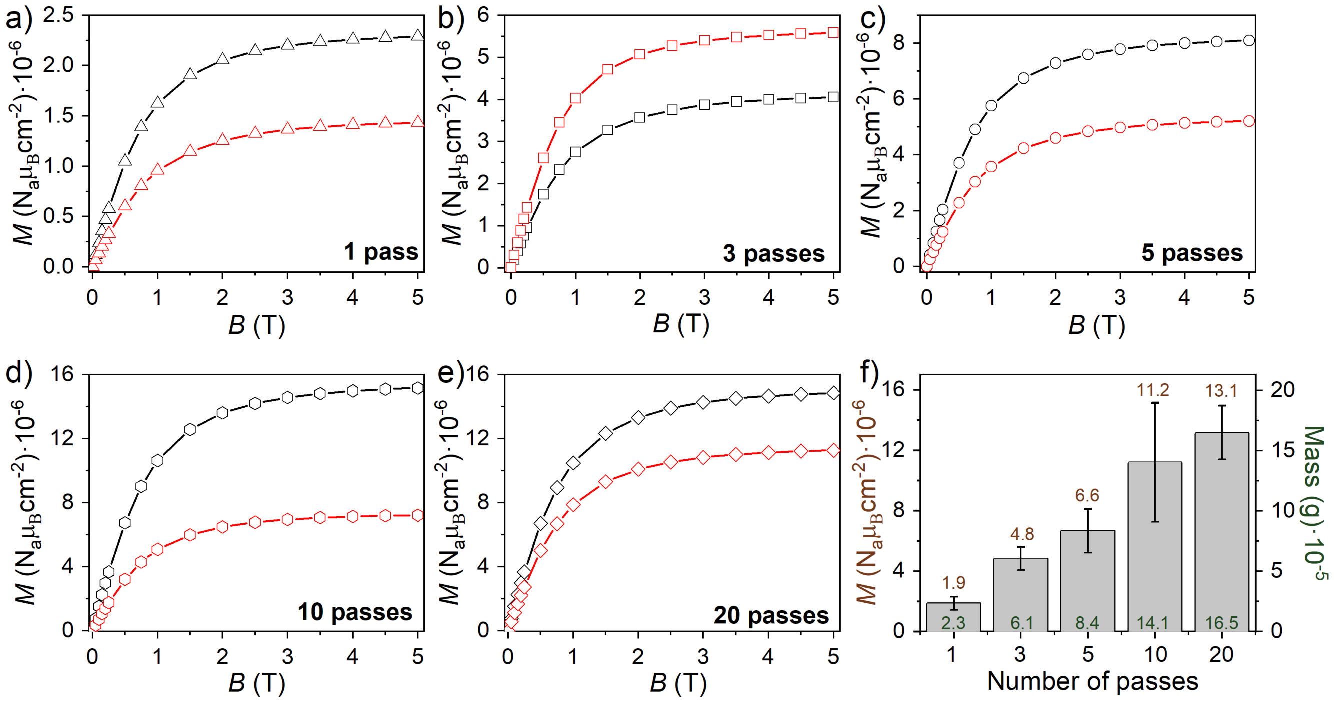

Figure S5. Isothermal surface magnetization at 2 K for deposits with increasing number of AJP passes: a) 1 pass; b) 3 passes; c) 5 passes; d) 10 passes; e) 20 passes; f) corresponding *M* at 5 T and calculated mass deposited. Error bars correspond to the deviation between 2 or 3 samples. All data correspond to deposits prepared with the Si substrate at 50ºC.

Table S1. Calculated mass of deposit per area of Si substrate as derived from the scaling of the *M vs. B* and χ *vs. T* data for 1-20 AJP passes as well as from the scaling of heat capacity data for a 20 passes deposit.

	**Mass (mg/cm^2)**		
	***M vs. B* data**	**χ *vs. T* data**	**C_p data**
**1 pass**	0.096	0.098	-
**3 passes**	0.17	0.17	-
**5 passes**	0.22	0.22	-
**10 passes**	0.63	0.64	-
**20 passes**	0.62	0.62	0.61

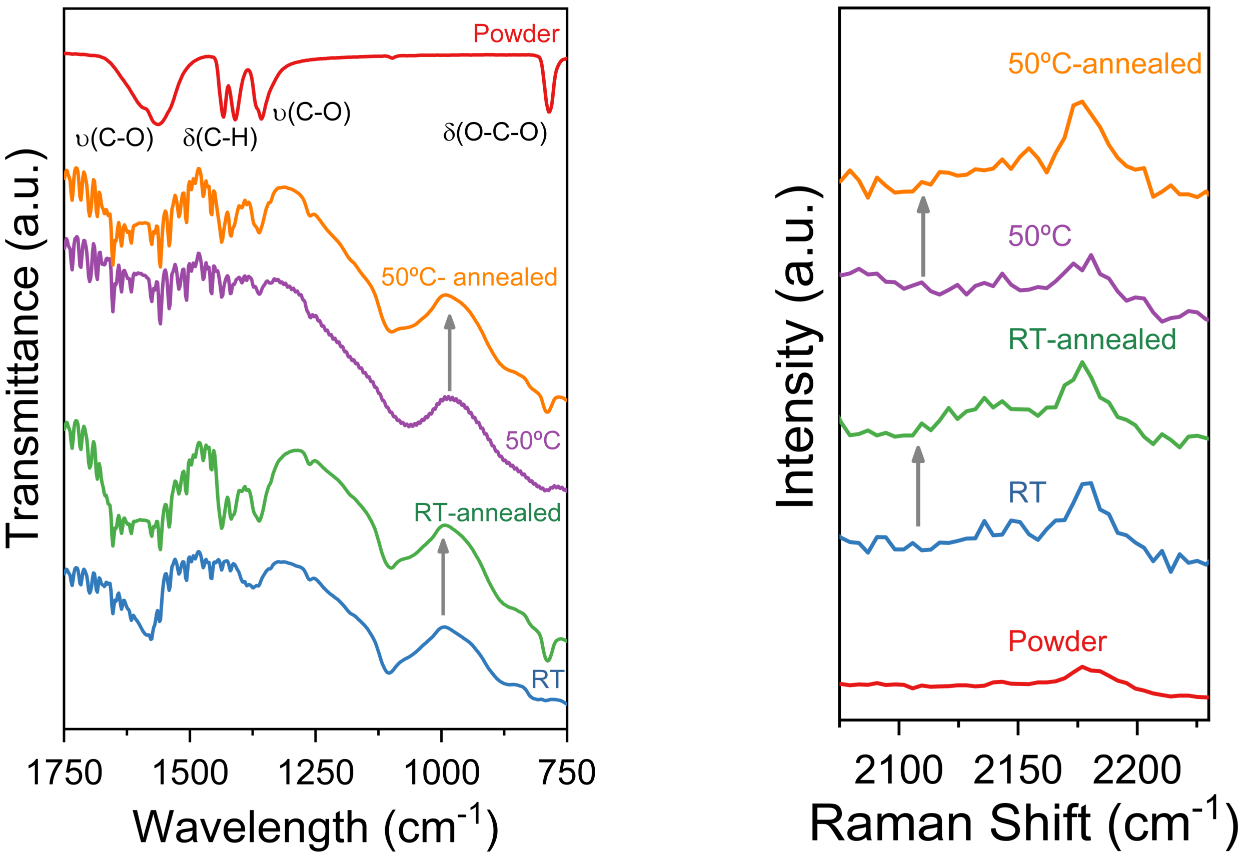

Figure S6. Left: Infra-red spectra of AJP deposits obtained with the Si substrate at RT and 50ºC, as obtained and after annealing at 80ºC, compared with the bulk powder spectrum. Right: Zoom of a weak band present in the Raman spectra of AJP deposits on Si, as initially obtained and after annealing at 80°C, compared with the bulk powder spectrum.

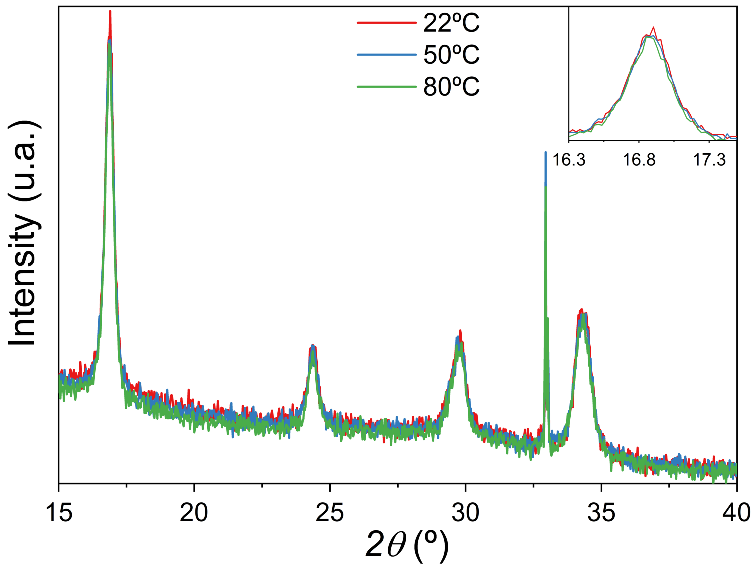

Figure S7. GIXRD patterns upon annealing a 10 passes AJP deposit prepared with the Si substrate at 50ºC. Measurements were performed continuously upon heating from RT (22ºC). Inset: Zoom of the most intense peak showing no variations in intensity or width.

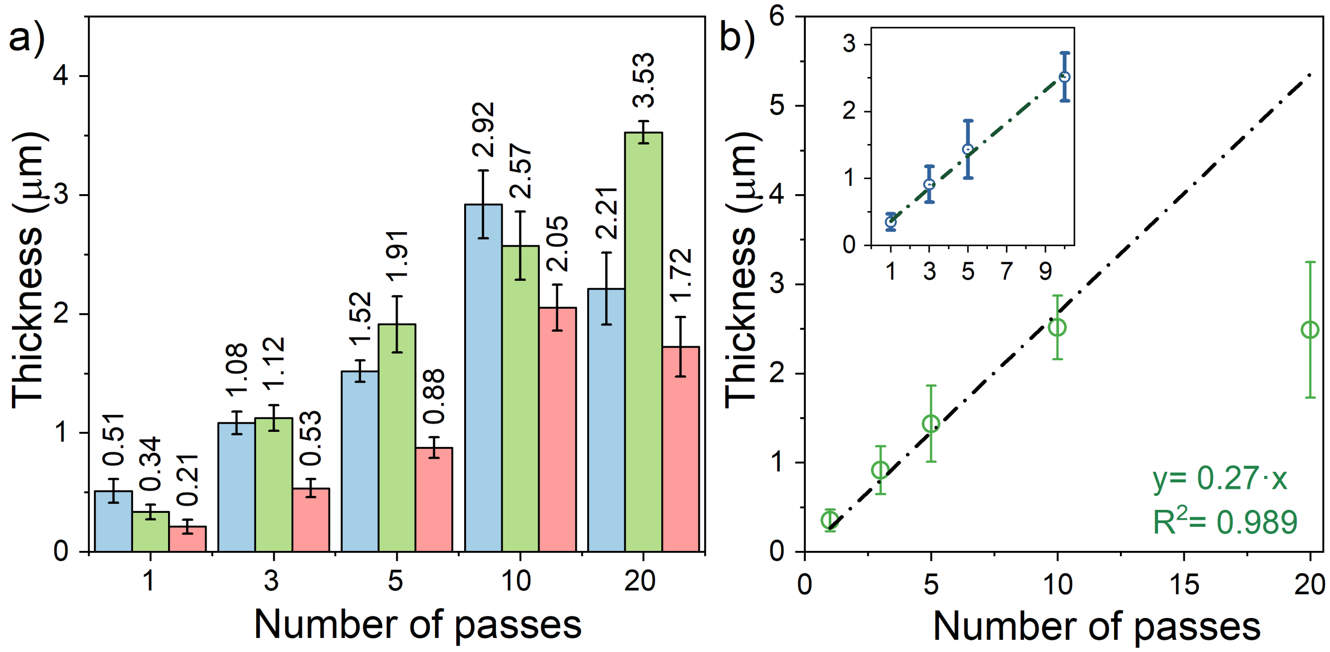

Figure S8. a) Thickness of deposits made with 1 to 20 AJP passes as derived from SEM images. b) Average thickness *vs.* number of passes. The dashed line is a linear regression giving a 0.27 μm/pass slope. The inset shows the same data up to 10 passes. Error bars correspond to the deviation between at least 3 samples. All data correspond to deposits prepared with the Si substrate at 50ºC.

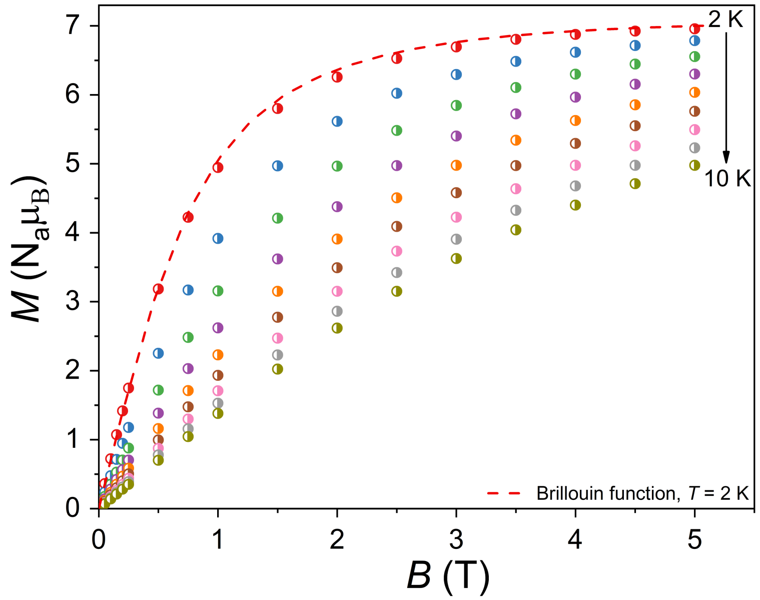

Figure S9. Isothermal field dependences in the 2-10 K range for a 5 AJP passes deposit. The raw data is obtained after subtraction of the diamagnetic component corresponding to the Si wafer, determined experimentally, and then scaled to reach the value calculated according to Brillouin function for an *S* = 7/2 spin and g = 2.02 at the highest field of 5 T. This gives the magnetization in Bohr magneton units $N_A\mu_B$. The dashed line is the Brillouin function for *S* = 7/2, g = 2.02 and *T* = 2 K.

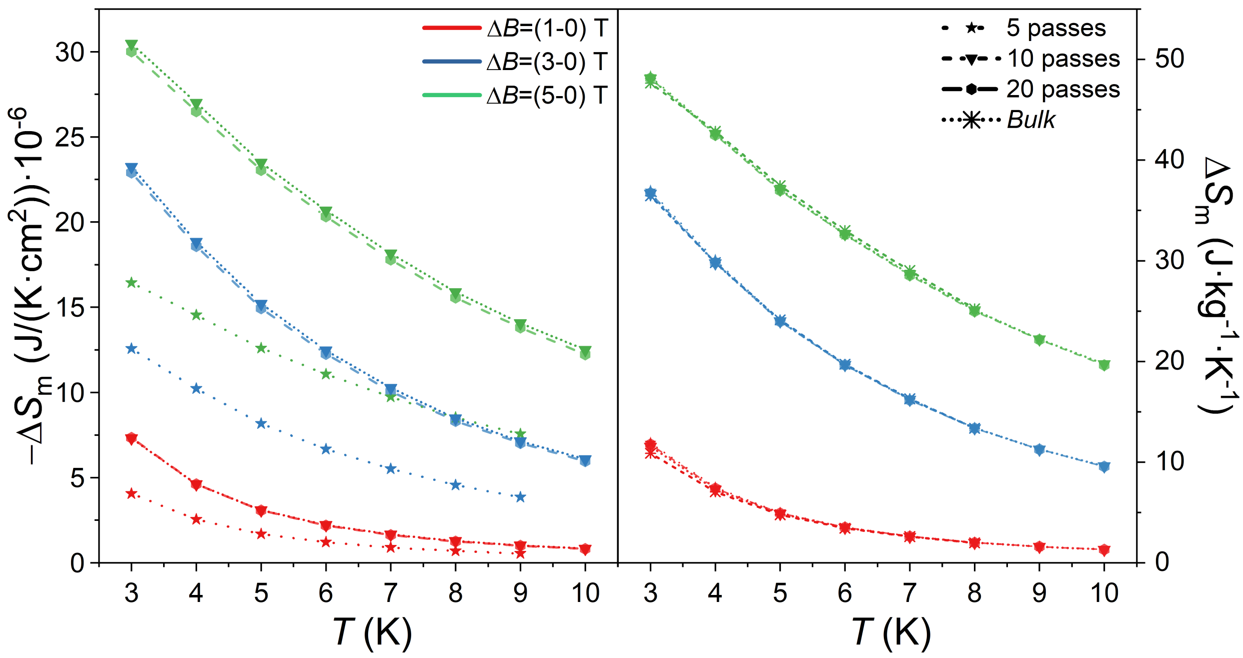

Figure S10. a) temperature dependence of the change in magnetic entropy ΔS_m expressed in surface units of deposits with 5, 10 and 20 AJP passes for different changes in the applied magnetic field ΔB. These are obtained from magnetization data such as those in Figure S9 and considering the equation $\boldsymbol{\Delta S_m(T, \Delta B)} = \int_{B_i}^{B_f} \left(\frac{\partial M(T,B)}{\partial T}\right)_B dB$. b) the same data expressed in mass units compared with those of the bulk material, showing an excellent agreement.

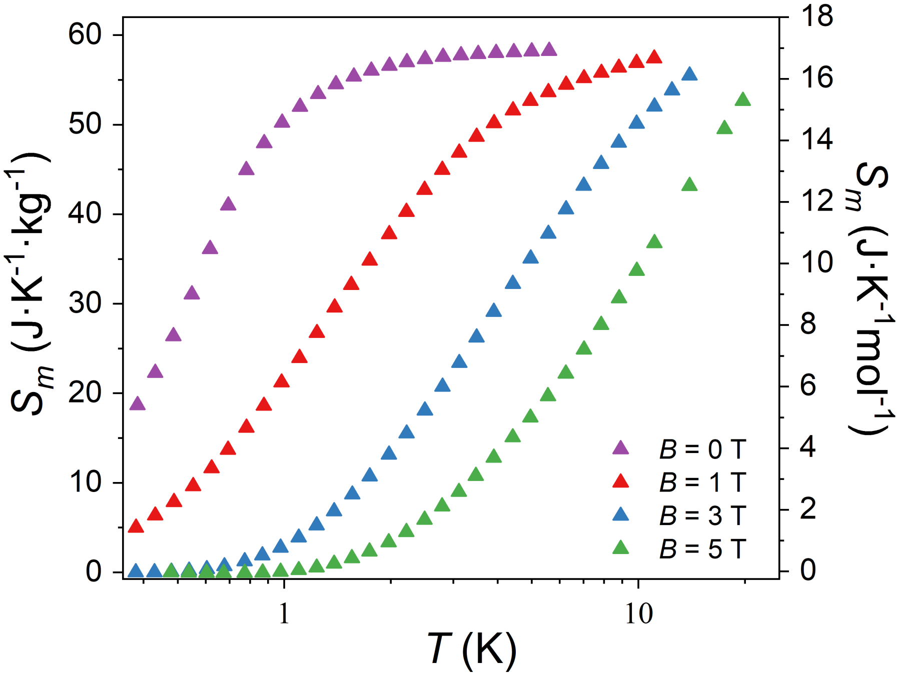

Figure S11. Temperature dependence of the experimental magnetic entropy, S_m, for several magnetic fields B, as obtained by integration of the C_m *vs.* T of a 20 AJP passes deposit.

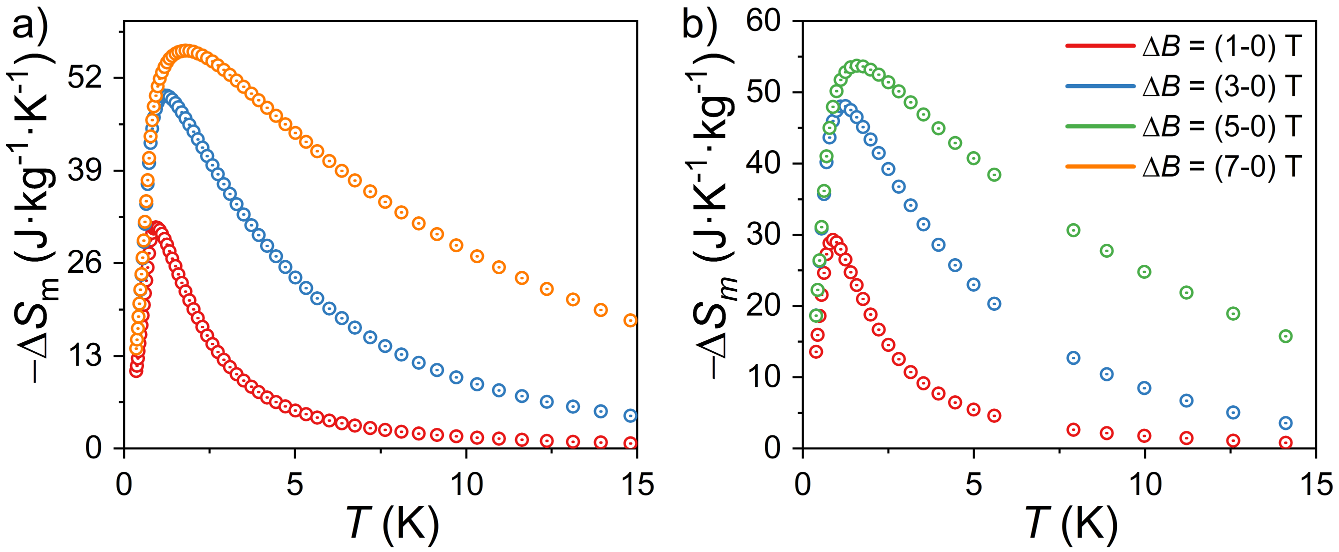

Figure S12. Temperature dependence of the change in magnetic entropy ΔS_m for various changes in magnetic field ΔB of the bulk $Gd(HCOO)_3$ material (left, a)), and of a 20 AJP passes deposit (right, b)) as obtained from the S_m data in Figure S11.

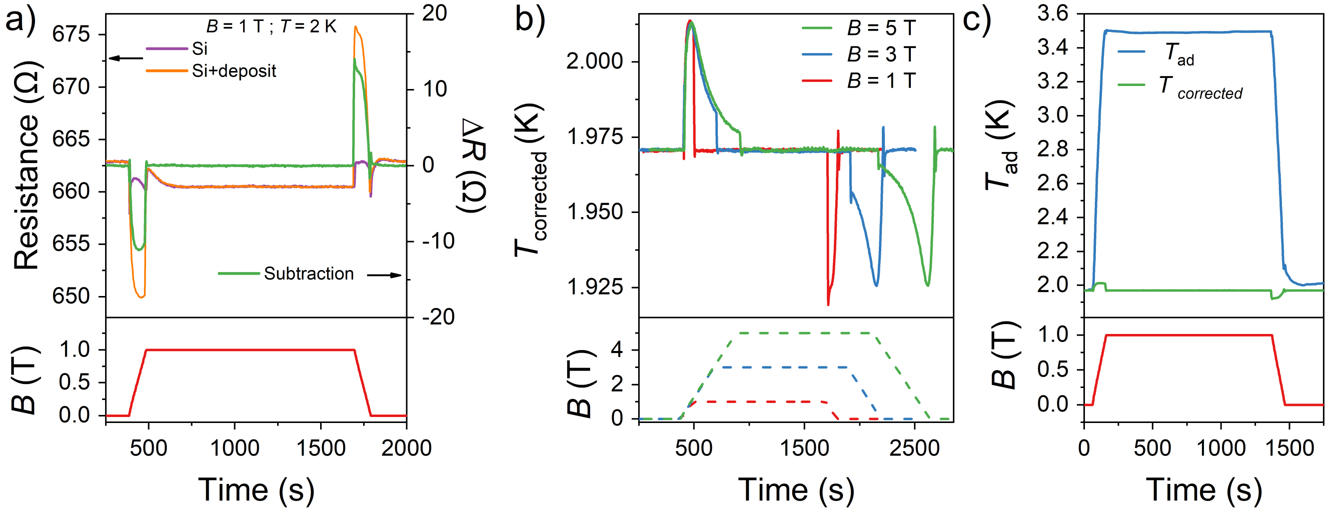

Figure S13. a) time evolution of the thermometer resistance upon the full magnetization-demagnetization cycle to 1 T for a 20 AJP passes deposit on Si (200 μm, —) and the same pristine piece of Si (—). The difference is given as green line (—) and shows the effect of the sole deposit, correcting magneto-resistive effects of the experimental set-up. b) Corrected measured temperature for full magnetization-demagnetization cycles to 1, 3 and 5 T, as derived from resistance measurements *vs.* time and calibration of the thermometer. The measured ΔT are small due to non-adiabatic conditions resulting from the connection of the set-up to thermal bath through metallic wires (see Scheme S2 for a description of the set-up). c) the time evolution of T_{ad}, the temperature that would be reached under ideal adiabatic temperature during a full magnetization-demagnetization cycle to 1 T and 2 K bath temperature. T_{ad} is obtained numerically from the measured T, by estimating the entropy losses/gains ΔS to/from the thermal bath, calculated as $\kappa(T-T_{bath})$ using the known wires thermal conductance $\kappa(T)$, and considering that $\boldsymbol{\Delta S} = \int_{T_{ad}}^{T} \frac{C}{T} \boldsymbol{dT}$, where C is the as-measured total heat capacity for the whole system {deposit+Si+sapphire platform}.[3] All magnetization and demagnetization cycles were done at 100 Oe/s sweep rate.

---

[3] A more detailed description of the procedure can be found in a) Sharples, J. W.; Collison, D.; McInnes, E. J. L.; Schnack, J.; Palacios, E.; Evangelisti, M. Quantum Signatures of a Molecular Nanomagnet in Direct Magnetocaloric Measurements. *Nature Commun.* **2014**, *5*, 5321. b) Palacios, E.; Sáez-Puche, R.; Romero, J.; Doi, Y.; Hinatsu, Y.; Evangelisti, M. Large Magnetocaloric effect in $EuGd_2O_4$ and $EuDy_2O_4$. *J. Alloys Cpds.* **2021**, *890*, 161847

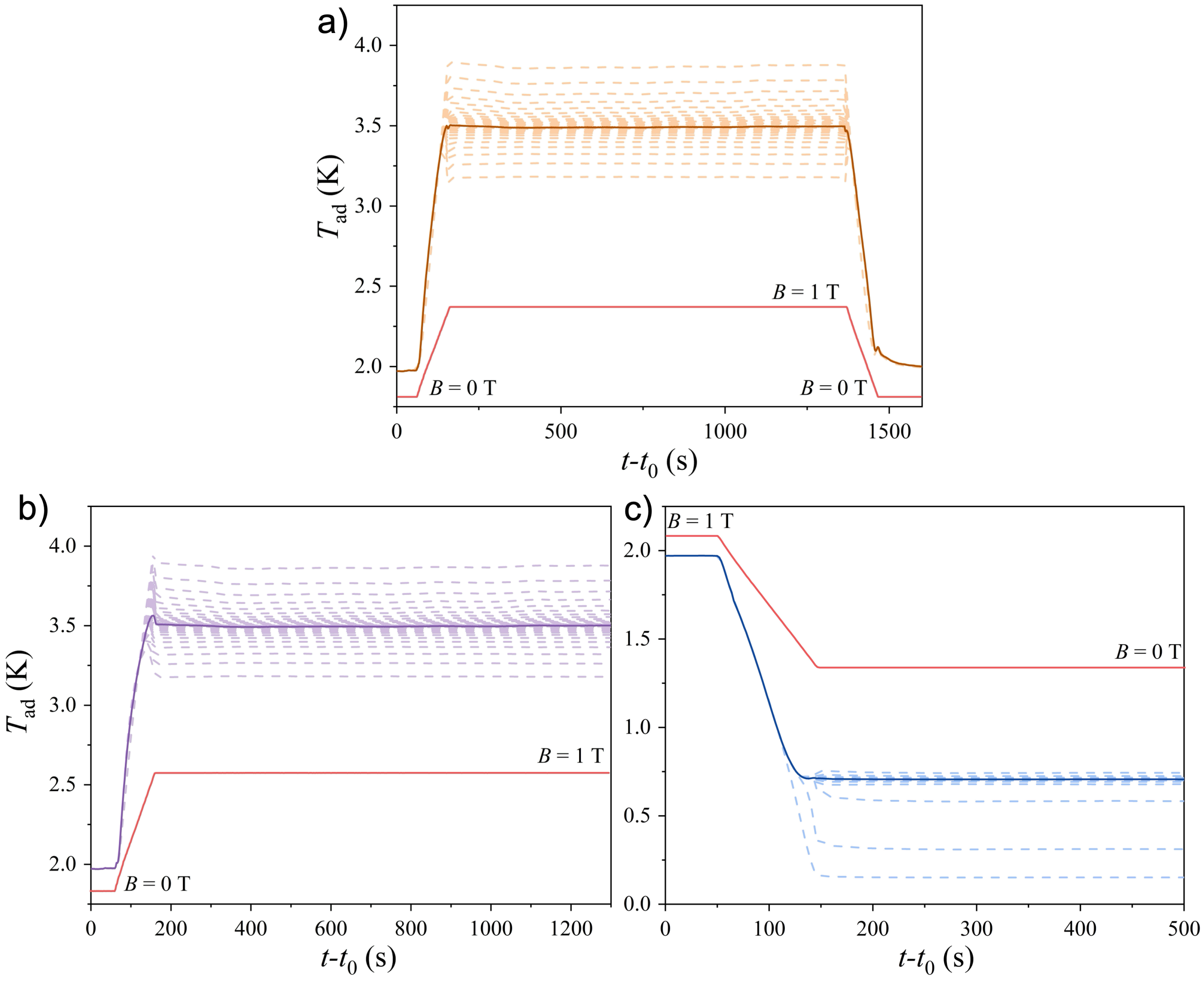

Figure S14. a) Time evolution of T_{ad}, the temperature that would be reached under ideal adiabatic temperature during a full magnetization-demagnetization cycle to 1 T and at 2 K bath temperature showing as dashed lines the successive steps in the numerical estimation of T_{ad}. Full lines depict the data once convergence has been reached, also shown in Figure S13c. b) and c) show the same data albeit treating separately the magnetization and demagnetization steps, *i.e.* in both cases starting from 2 K. In all cases T_{ad} is obtained numerically from the measured T shown in Figure S13b, by estimating the entropy losses/gains ΔS to/from the thermal bath, calculated as $\kappa(T\text{-}T_{bath})$ using the known wires thermal conductance $\kappa(T)$, and considering that $\boldsymbol{\Delta S} = \int_{T_{ad}}^{T} \frac{C}{T} dT$, where C is the as-measured total heat capacity for the whole system {deposit+Si+sapphire platform}.[4] All magnetization and demagnetization cycles were done at 100 Oe/s sweep rate.
